# Active Surveillance of Adverse Events Following Influenza Immunization in Jiangsu Province, China: A 2019–2023 Retrospective Study

**DOI:** 10.3390/vaccines13111154

**Published:** 2025-11-11

**Authors:** Zhiguo Wang, Sufang Wu, Xun Li, Ran Hu, Jing Yu, Borong Xu, Yuanyuan Zhu, Poning Liu

**Affiliations:** 1 Jiangsu Provincial Center for Disease Control and Prevention, Department of Expanded Program on Immunization, Nanjing 210009, China; njwang1718@163.com (Z.W.); 15706296973@163.com (X.L.);; 2 School of Public Health, Nanjing Medical University, Nanjing 211166, China; sufangwu0105@stu.njmu.edu.cn; 3 Department of Hematology and Oncology, Children’s Hospital of Nanjing Medical University, Nanjing 210093, China

**Keywords:** influenza vaccine, adverse events following immunization, the post-marketing safety study

## Abstract

**Background:** Influenza vaccines have been administered in Jiangsu Province. This study aimed to conduct a comprehensive retrospective analysis of influenza vaccine safety in the region from 2019 to 2023. **Methods:** Data were sourced from the Chinese National Adverse Events Following Immunization Information System (CNAEFIS) and Jiangsu Provincial Electronic Immunization Registries System (JSEIRS) systems. A comprehensive retrospective analysis was performed to calculate the incidence rates of adverse events following immunization (AEFI) and to identify potential safety signals through disproportionality analysis. **Results:** Out of 4,906,905 administered doses, 2080 AEFI cases were reported, yielding an overall incidence rate of 42.39 per 1,000,000 doses. Significantly higher rates were observed in children aged 6–35 months (71.03 per 1,000,000) and among recipients of trivalent vaccines (52.79 per 1,000,000) compared to quadrivalent vaccines (36.03 per 1,000,000). The vast majority of AEFIs were mild, common adverse reactions (94.47%, predominantly fever and local reactions), occurring predominantly within one day post-vaccination, while disproportionality analysis identified expected signals for common adverse reactions and rare local purulent infections. **Conclusions:** Overall, the findings affirm the vaccine’s favorable safety profile, align with pre-marketing data, and underscore the critical role of continuous post-marketing surveillance in maintaining public confidence and monitoring the safety of both established and new vaccine formulations.

## 1. Introduction

Influenza (commonly known as “flu”) is an acute respiratory infectious disease caused by influenza viruses. Recognized by the World Health Organization (WHO) as a top-priority globally monitored communicable disease [1], influenza spreads rapidly with extensive transmission range. Globally, it affects approximately 5–10% of adults and 20–30% of children annually, resulting in 3–5 million severe cases and 290,000–650,000 respiratory deaths [2]. As a populous country, China bears a substantial influenza-related morbidity and mortality burden. During 2011–2019, influenza caused an estimated economic loss of USD 4.6 to 14.9 billion annually in China [3], underscoring the urgent need for effective control measures.

Influenza viruses are classified into types A, B, C, and D based on nucleoprotein and matrix protein differences, with types A (Influenza A virus) and B (Influenza B virus) being the primary human pathogens. Influenza vaccines demonstrate 40–60% effectiveness [4], representing the most critical intervention for preventing infection and severe complications.

Currently, China’s licensed influenza vaccines include trivalent and quadrivalent formulations. The trivalent influenza vaccine consists of the trivalent inactivated influenza vaccine (IIV3)—which includes split-virion and subunit types—and the trivalent live attenuated influenza vaccine (LAIV3). These target two influenza A strains (H3N2 and H1N1) and one B strain (Victoria lineage). The quadrivalent inactivated vaccine (IIV4), including the split-virion and subunit versions, adds protection against a second B strain (Yamagata lineage).

Vaccine safety serves as the cornerstone for widespread immunization programs. While clinical trials have demonstrated acceptable safety profiles for licensed influenza vaccines [5,6,7], post-marketing surveillance remains essential to monitor potential adverse events following immunization in real-world populations. Given the antigenic variability of influenza viruses and the consequent need for annual vaccine updates, continuous adverse events following immunization (AEFI) monitoring plays a pivotal role in both maintaining public confidence in vaccination programs and ensuring comprehensive safety evaluations of updated vaccine formulations.

This study utilized data from the Chinese National Adverse Events Following Immunization Information System (CNAEFIS) to conduct a comprehensive analysis of influenza vaccine-associated AEFIs reported in Jiangsu Province during 2019–2023. The objectives were to characterize the occurrence and distribution of influenza vaccine-related AEFIs in Jiangsu Province and to provide data to inform subsequent post-marketing safety evaluations.

## 2. Methods

### 2.1. Data Sources

Based on the CNAEFIS and Jiangsu Provincial Electronic Immunization Registries System (JSEIRS), data on AEFI associated with influenza vaccines marketed and administered in Jiangsu Province from 2019 to 2023 were collated and analyzed, with data collection completed by 28 September 2023.

Data on influenza vaccination doses and concurrent doses of other vaccines administered in Jiangsu Province during the same period were obtained from the Jiangsu Provincial Electronic Immunization Registries System (JSEIRS) [8].

### 2.2. Categorization of AEFIs

Symptoms and diagnoses of AEFI cases were coded using the International Classification of Diseases, 10th Revision (ICD-10) [9]. AEFIs are classified into five types: type 1, vaccine product-related reactions, including common adverse reactions and rare adverse reactions; type 2, vaccine quality defect-related reactions; type 3, immunization error-related reactions; type 4, coincidental events; and type 5, immunization anxiety-related reactions [10].

### 2.3. Influenza Vaccine

Influenza vaccines in China are classified as non-National Immunization Program (NIP) vaccines. In Jiangsu Province, the available influenza vaccines include all types approved for national use: trivalent split-virion influenza vaccine, freeze-dried live attenuated influenza vaccine (nasal spray), and quadrivalent split-virion influenza vaccine. According to the Chinese Influenza Vaccination Technical Guidelines, the trivalent split-virion influenza vaccines are administered in two formulations: 0.25 mL/dose for children aged 6–35 months and 0.5 mL/dose for individuals aged ≥3 years. The freeze-dried live attenuated influenza vaccine (nasal spray, 0.20 mL/dose) is recommended for individuals aged 3–17 years. The quadrivalent split-virion influenza vaccines include three formulations: 0.25 mL/dose for children aged 6–35 months, 0.5 mL/dose for those aged ≥6 months, and 0.5 mL/dose for individuals aged ≥3 years. With the exception of the freeze-dried live attenuated influenza vaccine (nasal spray), which is administered via nasal inhalation, all other influenza vaccines are administered via intramuscular injection.

### 2.4. Data Analysis

Statistical analyses were performed using SPSS 26.0 (IBM SPSS Statistics 26). We conducted descriptive statistics on demographic characteristics (age, sex, region), vaccine types, AEFI classifications, clinical diagnoses, and time intervals between vaccination and AEFI onset. Temporal trends in AEFI reporting rates were visualized using line graphs. Comparative analyses of AEFI reporting rates employed chi-square tests or Fisher’s exact tests, with statistical significance set at *p* < 0.05.

Disproportionality analysis (DPA) was used to analyze influenza vaccine AEFIs, employing the following specific indicators: Proportional Reporting Ratio (PRR), Reporting Odds Ratio (ROR), Bayesian Confidence Propagation Neural Network (BCPNN), and the Medicines and Healthcare Products Regulatory Agency (MHRA) method. A signal was considered to be present if PRR 95% confidence interval lower limit (PRR_L0.05_) > 1; ROR 95% confidence interval lower limit (ROR_L0.05_) > 1; BCPNN Information Component (IC) 95% confidence interval lower limit (IC_L0.05_) > 0, and AEFI diagnosis cases ≥ 3, PRR ≥ 2, χ^2^ ≥ 4 (MHRA). These criteria indicate that the AEFI reporting rate for the influenza vaccine was higher than the average level for other vaccines.

## 3. Results

### 3.1. General Characteristics of AEFIs

From 1 January 2019 to 28 September 2023, a total of 4,906,905 doses of influenza vaccines were administered in Jiangsu Province, including 936,842 doses of trivalent split-virion influenza vaccine (for 6–35 months), 837,996 doses of trivalent split-virion influenza vaccine (for ≥3 years), 87,409 doses of freeze-dried live attenuated influenza vaccine (nasal spray), 6343 doses of quadrivalent split-virion influenza vaccine (for 6–35 months), 2,895,900 doses of quadrivalent split-virion influenza vaccine (for ≥3 years), and 142,415 doses of quadrivalent split-virion influenza vaccine (for ≥6 months). The CNAEFIS recorded 2080 AEFI cases, yielding an overall incidence rate (IR) of 42.39/1,000,000 doses (Table 1).

As shown in Table 1, statistical analysis revealed significant differences in AEFI incidence rates (IRs) across age groups and vaccine types (*p* < 0.05). The highest IRs were observed in children aged 6–35 months (71.03/1,000,000 doses) and recipients of quadrivalent split-virion influenza vaccine (for 6–35 months) (94.59/1,000,000 doses).

The highest number of AEFI cases was associated with the quadrivalent (split, ≥3 years) vaccine (*n* = 1003, IR = 34.64/1,000,000 doses), followed by the trivalent (split, 6–35 months) vaccine (*n* = 647, IR = 69.06/1,000,000 doses). Notably, the freeze-dried live attenuated (nasal spray) vaccine exhibited the highest IR among all vaccine types (IR = 94.59/1,000,000 doses), although based on a limited number of reports (*n* = 6) (Table 1).

### 3.2. Characteristics of AEFIs After Influenza Vaccine

As shown in Table 2, annual trends indicated fluctuations in AEFI reporting. The trivalent (split, 6–35 months) vaccine showed the highest IR in 2020 (IR = 84.35/1,000,000 doses), while the quadrivalent (split, ≥3 years) vaccine recorded its peak IR in 2019 (IR = 53.88/1,000,000 doses). The newly introduced quadrivalent (split, ≥6 months) vaccine in 2023 demonstrated an IR of 60.39/1,000,000 doses.

By reaction type, common adverse reactions constituted the vast majority of reports, with the highest IR again observed for the quadrivalent (split, 6–35 months). Abnormal reactions were infrequent, and very few cases of coincidental events were identified. No immunization anxiety-related reactions were reported (Table 2).

The number of reports for intervals between vaccination and AEFI onset of ≤1 day, 2–3 days, 4–7 days, 8–14 days, and ≥15 days were 1875 (90.14%), 176 (8.46%), 23 (1.11%), 5 (0.24%), and 1 (0.05%), respectively. The IRs for AEFI onset within ≤1 day were 62.12, 33.29, 40.04, 94.59, 31.04, and 51.96 per 1000,000 doses for the trivalent split-virion influenza vaccine (for 6–35 months), trivalent split-virion influenza vaccine (for ≥3 years), freeze-dried live attenuated influenza vaccine (nasal spray), quadrivalent split-virion influenza vaccine (for 6–35 months), quadrivalent split-virion influenza vaccine (for ≥3 years), and quadrivalent split-virion influenza vaccine (for ≥6 months), respectively. The time interval for reaction onset was primarily concentrated within ≤1 day. (Table 2)

The surveillance analysis revealed significant differences in AEFI reporting patterns across vaccine classifications (Table 3). Among the AEFI reports, trivalent influenza vaccines accounted for 985 cases (47.36%), while quadrivalent influenza vaccines accounted for 1095 cases (52.64%). The IR for trivalent influenza vaccines was 52.89/1000,000 doses, which was significantly higher than the IR for quadrivalent influenza vaccines (*p* < 0.001). Based on the recommended dosage per injection, the vaccines were classified into 0.20 mL, 0.25 mL, and 0.5 mL dose groups. The 0.20 mL dose group accounted for 41 cases (1.97%), the 0.25 mL dose group accounted for 653 cases (31.39%), and the 0.5 mL dose group accounted for 1. The 0.25 mL dose group exhibited the highest IR (*p* < 0.001), at 69.23/1,000,000 doses. Based on the administration route, influenza vaccines were classified into intramuscular injection and nasal inhalation. The intramuscular injection group accounted for 2039 cases (98.03%), while the nasal inhalation group accounted for 41 cases (1.97%).

### 3.3. Symptoms and Diagnosis

Table 4 presents the symptoms and diagnostic features associated with different influenza vaccines. Among the AEFI reports, fever, local redness, and local induration were commonly reported symptoms. For fever, 97 cases (2.84%) reported a temperature of 37.1–37.5 °C, 655 cases (19.19%) reported a temperature of 37.6–38.5 °C, and 746 cases (21.86%) reported a temperature ≥ 38.6 °C. The highest IR was observed for fever ≥ 38.6 °C (15.16/1,000,000 doses), with the trivalent split-virion influenza vaccine (for 6–35 months) exhibiting the highest IR (32.02/1,000,000 doses) for this symptom.

For local redness, 158 cases (4.63%) reported a size ≤ 2.5 cm, 265 cases (7.76%) reported a size of 2.6–5.0 cm, and 106 cases (3.11%) reported a size > 5.0 cm. The highest IR was observed for local redness and swelling of 2.6–5.0 cm (5.39/1,000,000 doses), with the quadrivalent split-virion influenza vaccine (for ≥6 months) exhibiting the highest IR (10.53/1,000,000 doses) for this symptom (Table 4).

For local induration, 92 cases (2.70%) reported a size ≤ 2.5 cm, 128 cases (3.75%) reported a size of 2.6–5.0 cm, and 45 cases (1.32%) reported a size > 5.0 cm. The highest IR was observed for local induration of 2.6–5.0 cm (2.60/1,000,000 doses), with the quadrivalent split-virion influenza vaccine (for ≥6 months) exhibiting the highest IR (4.92/1,000,000 doses) for this symptom (Table 4).

Among other symptoms, fatigue, crying, and loss of appetite were reported with relatively high IRs of 4.49, 4.25, and 2.60/1,000,000 doses, respectively. Specifically, the freeze-dried live attenuated influenza vaccine (nasal spray) exhibited the highest IR for fatigue (5.72/1,000,000 doses), the trivalent split-virion influenza vaccine (for 6–35 months) exhibited the highest IR for crying (13.88/1,000,000 doses), and the quadrivalent split-virion influenza vaccine (for ≥6 months) exhibited the highest IR for loss of appetite (4.92/1,000,000 doses) (Table 4).

### 3.4. Final Clinical Diagnosis and Disproportionality Analysis

Among the final clinical diagnoses, general reaction symptoms (fever/redness/induration, etc.) were the most common, reported in 1965 cases (94.47%), with an IR of 40.05/1,000,000 doses. The IRs for other clinical diagnoses ranged from 0.02 to 0.98 per 1,000,000 doses. Disproportionality analysis revealed a ROR of 1.71 (95% CI: 1.41, 2.07) for general reaction symptoms (fever/redness/induration, etc.) in the final clinical diagnoses of AEFI, indicating a positive signal. Local purulent infections—lymphangitis and lymphadenitis—had a PRR of 22.16 (95% CI: 2.01, 244.55) and a ROR of 22.17 (95% CI: 2.01, 244.56), also indicating a positive signal (Table 5).

### 3.5. Analysis of Factors Associated with Common Adverse Reactions

Statistically significant differences were observed in the proportional distribution of reported fever, local redness/swelling, and local induration across age groups (*p* < 0.001). The 3–17 years age group had the highest number of reports for fever (832 cases, 40%), local redness/swelling (299 cases, 14.38%), and local induration (142 cases, 6.83%). Significant gender-based differences were noted in the proportion of fever reports (*p* < 0.001), with males and females reporting 741 cases (35.63%) and 757 cases (36.39%), respectively. The quadrivalent split-virion influenza vaccine (for ≥3 years) showed higher reported frequencies of fever (644 cases, 30.96%), local redness/swelling (337 cases, 16.20%), and local induration (167 cases, 8.03%) compared to other influenza vaccines (*p* < 0.001). The majority of fever (1379 cases, 66.30%) and local redness/swelling (458 cases, 22.02%) occurred within ≤1 day post-vaccination, with statistically significant differences in onset timing (*p* < 0.001) (Table 6).

## 4. Discussion

In this study’s AEFI reports, the 6–35 months age group had the highest AEFI IR. The quadrivalent split-virion influenza vaccine (for 6–35 months) had the highest AEFI IR. The trivalent split-virion influenza vaccine (for 6–35 months) had a relatively high AEFI IR. A Canadian influenza vaccine surveillance study indicated that the IR of vaccine-related health events in the 6-month to 4-year age group was higher than in other age groups [11]. AusVaxSafety (Australian Vaccine Safety Surveillance System) also reported a higher AEFI IR in the 6-month to 4-year age group [12]. This may be because the immune systems of children aged 6–35 months are not fully developed, and many are receiving their first influenza vaccination [13].

The 6–35 months age group had the highest IR in 2020, while the 3–17 years and 18–49 years age groups had the highest IR in 2019. The trivalent split-virion influenza vaccine (for 6–35 months) had the highest IR in 2020, the trivalent split-virion influenza vaccine (for ≥3 years) had the highest IR in 2022, and the quadrivalent split-virion influenza vaccine (for ≥3 years) had the highest IR in 2019. These differences were statistically significant. The differences in AEFI IR across different years may be related to the batch-to-batch consistency of vaccine administered in those years. During the COVID-19 pandemic, people also received various types of COVID-19 vaccines. Additionally, fever, headache, and other symptoms caused by COVID-19 infection or other illnesses may have been mistakenly attributed to influenza vaccination.

In the analysis of AEFI reports by different seasons, April–June had the highest AEFI IR, and January–March had a relatively high IR, but these differences were not statistically significant. Influenza occurs throughout the year, mainly concentrated in the winter and spring seasons. Studies have shown that Jiangsu Province, as a southern Chinese city, has a consistently high incidence of influenza in winter and spring [14]. The different IRs in different regions may be due to differences in the sensitivity of AEFI surveillance in those regions, or they may be related to the region’s economic level and healthcare conditions. The seasonal distribution, annual trends, and type-specific reporting characteristics of influenza vaccine AEFI incidence rates in Jiangsu Province from 2019 to 2023 are detailed in the Supplementary Figure S1.

AEFIs are classified into five types based on the cause: adverse reactions, vaccine quality incidents, vaccination accidents, coincidental events, and immunization anxiety related reaction. Adverse reactions are further divided into common adverse reactions and rare adverse reactions. This study found that the quadrivalent split-virion influenza vaccine (for 6–35 months) had the highest IR for common adverse reactions, and the trivalent split-virion influenza vaccine (for 6–35 months) had the highest IR for rare adverse reactions. Overall, common adverse reactions were the predominant type of report for all vaccines, which is similar to previous studies, indicating that AEFIs from vaccines are mainly mild symptoms [15].

The AEFI IR for trivalent influenza vaccines was higher than that for quadrivalent influenza vaccines, which differs from a review study [16]. This may be related to differences in the production methods of vaccines administered in different regions, as well as regional differences in the level of AEFI reporting surveillance. The influenza vaccine with a dosage of 0.25 mL/vial had the highest AEFI IR. The administration of the 0.25 mL/vial dose is for children aged 6–35 months. Similar to the reasons mentioned above, the AEFI reports may be higher because children aged 6–35 months are not fully developed. The number of nasal spray vaccinations was small, and there was no statistically significant difference in reports between different routes of administration.

Among common adverse reactions, fever ≥ 38.6 °C had the highest IR, followed by local redness 2.6–5.0 cm and local induration 2.6–5.0 cm. Among other reactions, fatigue, and loss of appetite were common. The quadrivalent split-virion influenza vaccine (for 6–35 months) had the highest IR for fever reactions, and the quadrivalent split-virion influenza vaccine (for ≥6 months) had the highest IR for local redness and local induration reactions. Similarly to other studies, the main AEFIs after vaccination were fever, redness, and induration [17,18].

Disproportionality analysis can monitor adverse reaction signals of drugs/vaccines, effectively warn of the occurrence and development of adverse reactions of drugs/vaccines, thereby reminding of possible drug/vaccine safety risks, and provide a reliable basis for safe medication. This method has been widely used in drug/vaccine safety research [19,20]. The disproportionality analysis in this study showed positive ROR signals for general reaction symptom diagnoses (fever/redness/induration, etc.) and positive PRR and ROR signals for local purulent infection—lymphangitis and lymphadenitis, suggesting that compared with other vaccines in Jiangsu Province during the same period, influenza vaccination had a higher IR for general reaction symptom diagnoses (fever/redness/induration, etc.) and local purulent infection—lymphangitis and lymphadenitis, which should be given special attention. Nevertheless, the notable ROR and PRR signals for ‘Local Purulent Infection’ are derived from a solitary case accompanied by broad confidence intervals, suggesting limited robustness. Consequently, this finding should be regarded as a hypothesis-generating observation that necessitates validation in larger datasets. Previous studies have shown that influenza vaccination increases the reporting of Guillain–Barré syndrome (GBS) [21,22,23], but this study did not find positive signals for GBS reports. This observation merits significant emphasis, as it conveys a crucially reassuring message for public health. The absence of detectable severe adverse events following immunization (AEFI) signals within a large-scale, population-based surveillance system such as ours offers substantial empirical evidence concerning the safety of influenza vaccines administered in Jiangsu Province during the study period.

AEFI occurrence was mainly concentrated on the day of vaccination, similar to the AEFI monitoring results of influenza vaccines in Zhejiang Province [18]. This suggests that healthcare professionals should focus on the occurrence of adverse reactions within 1 day after vaccination. In the general reaction analysis, the IR of fever, local redness, and local induration in the 3–17 years age group was higher than in other age groups. This may be because as children get older, their self-expression abilities develop, they can better describe and identify abnormal physical conditions, and they are more likely to report adverse reactions [24]. The IR of fever was higher in females than in males. Studies have shown that AEFI reports are higher in females than in males [25]. Further research is needed to investigate the relationship between gender and AEFI reporting. The quadrivalent split-virion influenza vaccine (for ≥3 years) had a higher IR for fever, local redness, and local induration than other vaccines. The reasons for this need further investigation.

This study provides a basis for the first post-marketing safety assessment of the full-dose (0.5 mL) quadrivalent split influenza vaccine for people aged 6 months and above. The study also has limitations. Our study used passive surveillance data, which has certain limitations. For example, the vaccine safety awareness of vaccinees or their guardians, the completeness of reports by healthcare professionals, etc., will affect the quality of the reports. Passive surveillance only has information from AEFI reporters, so some conclusions in the article lack controls. More prospective active surveillance studies are needed in the future to confirm our results.

## 5. Conclusions

Our findings indicate that the AEFIs reported following influenza vaccination in Jiangsu Province from January 2019 to September 2023 were predominantly mild and common reactions. No new or unexpected safety concerns were identified, which is consistent with pre-marketing clinical trials. Continued monitoring of influenza vaccine safety by CNAEFIS is warranted, particularly for newly marketed vaccines.

## Figures and Tables

**Table 1:** General characteristics of influenza vaccine AEFIs in Jiangsu Province, 2019–2023.

		Total				χ^2^	*p*
		Total Doses	AEFI Cases	Proportion (%)	IR		
Total		4,906,905	2080	100	42.39		
Age Group	6–35 months	963,030	684	32.88	71.03	520.161	0.000
	3–17 years	2,307,563	1144	55.00	49.58		
	18–49 years	651,838	164	7.88	25.16		
	≥50 years	984,474	88	4.23	8.94		
Sex	Male	2,335,399	990	47.60	42.39	0.000	0.999
	Female	2,571,506	1090	52.40	42.39		
Vaccine Type	Trivalent (split, 6–35 months)	936,842	647	31.11	69.06	223.326	0.000
	Trivalent (split, ≥3 years)	837,996	297	14.28	35.44		
	Freeze-dried live (nasal spray)	87,409	41	1.97	46.91		
	Quadrivalent (split, 6–35 months)	6343	6	0.29	94.59		
	Quadrivalent (split, ≥3 years)	2,895,900	1003	48.22	34.64		
	Quadrivalent (split, ≥6 months)	142,415	86	4.13	60.39		

AEFIs: adverse events following immunization; IR: incidence rate (per 1,000,000 doses).

**Table 2:** Influenza vaccination and AEFI reports in Jiangsu Province from 2019 to 2023.

Total		Trivalent (Split, 6–35 Months)		Trivalent (Split, ≥3 Years)		Freeze-Dried Live (Nasal Spray)		Quadrivalent (Split, 6–35 Months)		Quadrivalent (Split, ≥3 Years)		Quadrivalent (Split, ≥6 Months)	
		AEFI Cases	IR	AEFI Cases	IR	AEFI Cases	IR	AEFI Cases	IR	AEFI Cases	IR	AEFI Cases	IR
Year													
	2019	76	48.59	60	29.16	–	–	–	–	106	53.88	–	–
	2020	220	84.35	103	29.63	13	69.93	–	–	212	30.42	–	–
	2021	179	73.49	51	41.93	7	41.98	–	–	181	27.77	–	–
	2022	156	70.76	67	53.87	18	51.46	–	–	345	37.99	–	–
	2023	16	28.78	16	41.49	3	17.47	6	94.59	159	35.94	86	60.39
Reaction Type													
	Common adverse reactions	618	65.97	285	26.01	40	45.76	6	94.59	963	33.32	84	58.98
	Rare adverse reactions	27	2.88	11	1.31	1	1.14	0	0.00	34	1.17	2	1.40
	Coincidental events	2	0.21	1	0.12	0	0.00	0	0.00	6	0.21	0	0.00
	Immunization anxiety-related reaction	0	0.00	0	0.00	0	0.00	0	0.00	0	0.00	0	0.00
Time Interval													
	≤1 day	582	62.12	279	33.29	35	40.04	6	94.59	899	31.04	74	51.96
	2–3 days	55	5.87	15	1.79	5	5.72	0	0.00	91	3.14	10	7.02
	4–7 days	10	1.07	3	0.36	1	1.14	0	0.00	7	0.24	2	1.40
	8–14 days	0	0.00	0	0.00	0	0.00	0	0.00	5	0.17	0	0.00
	≥15 days	0	0.00	0	0.00	0	0.00	0	0.00	1	0.03	0	0.00
Total		647	69.06	297	35.44	41	46.91	6	94.59	1003	34.64	86	60.39

Note: AEFIs: adverse events following immunization; IR: incidence rate (per 1,000,000 doses).

**Table 3:** Distribution of AEFI IR by influenza vaccine category in Jiangsu Province.

		AEFI Cases	%	IR	Χ^2^	*p*
Vaccine Type	Trivalent	985	47.36	52.89	78.150	0.000
	Quadrivalent	1095	52.64	35.96		
Dose (mL/vial)	0.2	41	1.97	46.91	201.087	0.000
	0.25	653	31.39	69.23		
	0.5	1386	66.63	35.76		
Administration Route	Intramuscular injection	2039	98.03	42.31	0.428	0.513
	Nasal	41	1.97	46.91		

Note: AEFIs: adverse events following immunization; IR: incidence rate (per 1,000,000 doses).

**Table 4:** Symptoms and diagnostic characteristics of AEFI after influenza vaccination in Jiangsu Province, 2019–2023.

		Trivalent (Split, 6–35 Months)		Trivalent (Split, ≥3 Years)		Freeze-Dried Live (Nasal Spray)		Quadrivalent (Split, 6–35 Months)		Quadrivalent (Split, ≥3 Years)		Quadrivalent (Split, ≥6 Months)		Total		
		AEFI Cases	IR	AEFI Cases	IR	AEFI Cases	IR	AEFI Cases	IR	AEFI Cases	IR	AEFI Cases	IR	AEFI Cases	%	IR
Fever (℃)																
	37.1–37.5	21	2.24	15	1.79	4	4.58	0	0.00	52	1.80	5	3.51	97	2.84	1.97
	37.6–38.5	228	24.34	99	11.81	16	18.30	2	31.53	283	9.77	27	18.96	655	19.19	13.31
	≥38.6	300	32.02	85	10.14	18	20.59	3	47.30	309	10.67	31	21.77	746	21.86	15.16
Local Redness (cm)																
	≤2.5	40	4.27	29	3.46	0	0.00	0	0.00	80	2.76	9	6.32	158	4.63	3.21
	2.6–5.0	30	3.20	32	3.82	0	0.00	0	0.00	188	6.49	15	10.53	265	7.76	5.39
	>5.0	12	1.28	22	2.63	0	0.00	1	15.77	69	2.38	2	1.40	106	3.11	2.15
Local Induration (cm)																
	≤2.5	21	2.24	18	2.15	0	0.00	0	0.00	47	1.62	6	4.21	92	2.70	1.87
	2.6–5.0	17	1.81	16	1.91	0	0.00	0	0.00	88	3.04	7	4.92	128	3.75	2.60
	>5.0	4	0.43	6	0.72	0	0.00	0	0.00	32	1.11	3	2.11	45	1.32	0.91
Other Symptoms																
	Sweating	6	0.64	1	0.12	0	0.00	0	0.00	5	0.17	0	0.00	12	0.35	0.24
	Nausea	3	0.32	4	0.48	1	1.14	0	0.00	14	0.48	0	0.00	22	0.64	0.45
	Fatigue	49	5.23	36	4.30	5	5.72	0	0.00	124	4.28	7	4.92	221	6.48	4.49
	Abdominal Pain	1	0.11	1	0.12	0	0.00	0	0.00	6	0.21	1	0.70	9	0.26	0.18
	Diarrhea	6	0.64	2	0.24	0	0.00	1	15.77	1	0.03	1	0.70	11	0.32	0.22
	Arthralgia	0	0.00	2	0.24	1	1.14	0	0.00	2	0.07	0	0.00	5	0.15	0.10
	Cough	11	1.17	7	0.84	3	3.43	0	0.00	26	0.90	4	2.81	51	1.49	1.04
	Myalgia	2	0.21	7	0.84	1	1.14	0	0.00	41	1.42	2	1.40	53	1.55	1.08
	Rhinorrhea	7	0.75	3	0.36	1	1.14	0	0.00	10	0.35	2	1.40	23	0.67	0.47
	Pallor	0	0.00	0	0.00	0	0.00	0	0.00	4	0.14	0	0.00	4	0.12	0.08
	Vomiting	24	2.56	5	0.60	1	1.14	0	0.00	32	1.11	0	0.00	62	1.82	1.26
	Rash	21	2.24	5	0.60	0	0.00	0	0.00	23	0.79	4	2.81	53	1.55	1.08
	Itching	12	1.28	8	0.95	0	0.00	0	0.00	40	1.38	2	1.40	62	1.82	1.26
	Poor Appetite	43	4.59	15	1.79	2	2.29	0	0.00	61	2.11	7	4.92	128	3.75	2.60
	Drowsiness	24	2.56	11	1.31	1	1.14	0	0.00	24	0.83	1	0.70	61	1.79	1.24
	Headache	0	0.00	12	1.43	0	0.00	0	0.00	42	1.45	1	0.70	55	1.61	1.12
	Dizziness	1	0.11	10	1.19	4	4.58	0	0.00	22	0.76	1	0.70	38	1.11	0.77
	Palpitations	0	0.00	1	0.12	0	0.00	0	0.00	1	0.03	0	0.00	2	0.06	0.04
	Chest Tightness	0	0.00	0	0.00	0	0.00	0	0.00	2	0.07	0	0.00	2	0.06	0.04
	Redness of Pharynx	0	0.00	1	0.12	0	0.00	0	0.00	4	0.14	0	0.00	5	0.15	0.10
	Crying	130	13.88	8	0.95	4	4.58	1	15.77	52	1.80	14	9.83	209	6.12	4.25
	Others	8	0.85	3	0.36	0	0.00	0	0.00	22	0.76	0	0.00	33	0.97	0.67
Total		1021	108.98	464	55.37	62	70.93	8	126.12	1706	58.91	152	106.73	3413	100.00	69.36

Note: AEFIs: adverse events following immunization; IR: incidence rate (per 1,000,000 doses).

**Table 5:** Final clinical diagnosis distribution of AEFI classifications after influenza vaccination in Jiangsu Province, 2019–2023.

Diagnosis	Total			PRR	PRR (95% CI)	ROR	ROR (95% CI)	MHRA (χ^2^)	IC (IC_L0.05_)
	AEFI Cases	%	IR						
Total	2080	100.00	42.39						
General Reaction (Fever/Redness/Induration, etc.)	1965	94.47	40.05	1.04	(0.98, 1.11)	1.71	(1.41, 2.07)	31.19	−0.15
Epilepsy	1	0.05	0.02	4.43	(0.57, 34.63)	4.43	(0.57, 34.65)	2.42	−4.41
Allergic Reaction—Maculopapular Rash	3	0.14	0.06	0.77	(0.25, 2.42)	0.77	(0.25, 2.42)	0.20	−3.65
Allergic Reaction—Allergic Rash	48	2.31	0.98	0.54	(0.40, 0.71)	0.52	(0.39, 0.70)	20.01	−1.84
Allergic Reaction—Allergic Purpura	2	0.10	0.04	1.28	(0.31, 5.24)	1.28	(0.31, 5.24)	0.12	−3.70
Allergic Reaction—Urticaria	12	0.58	0.24	0.58	(0.33, 1.02)	0.58	(0.33, 1.02)	3.67	−2.59
Allergic Reaction—Angioedema	2	0.10	0.04	1.09	(0.27, 4.45)	1.09	(0.27, 4.45)	0.02	−3.83
Allergic Reaction—Other Allergic Reactions	8	0.38	0.16	1.90	(0.93, 3.85)	1.90	(0.93, 3.86)	3.25	−1.51
Local Purulent Infection—Lymphangitis and Lymphadenitis	1	0.05	0.02	22.16	(2.01, 244.55)	22.17	(2.01, 244.56)	13.47	−4.88
Guillain–Barré Syndrome	1	0.05	0.02	1.43	(0.20, 10.48)	1.43	(0.20, 10.48)	0.13	−4.63
Febrile Convulsion	2	0.10	0.04	1.27	(0.31, 5.17)	1.27	(0.31, 5.17)	0.11	−3.72
Thrombocytopenic Purpura	1	0.05	0.02	0.59	(0.08, 4.25)	0.59	(0.08, 4.25)	0.28	−5.19
Other, Other Preliminary Clinical Diagnoses	32	1.54	0.65	0.53	(0.37, 0.76)	0.53	(0.37, 0.75)	13.34	−2.04
Blank	2	0.10	0.04						

Note: AEFIs: adverse events following immunization; IR: incidence rate (per 1,000,000 doses). This table presents the frequency distribution of AEFI cases by clinical diagnosis category and the results of the disproportionality analysis. Signal detection was performed using four metrics: Proportional Reporting Ratio (PRR), Reporting Odds Ratio (ROR), Bayesian Confidence Propagation Neural Network (BCPNN), and the Medicines and Healthcare products Regulatory Agency (MHRA) method. A safety signal was flagged if the following criteria were met for each metric: PRR: Lower limit of the PRR 95% confidence (PRRL0.05) > 1. ROR: Lower limit of the ROR 95% confidence interval (RORL0.05)) > 1. BCPNN: Lower limit of the 95% confidence interval for the Information Component (ICL0.05) > 0. MHRA: Number of AEFI cases ≥ 3, PRR ≥ 2, and χ^2^ ≥ 4.

**Table 6:** Correlation analysis of general reaction reporting after influenza vaccination in Jiangsu Province, 2019–2023.

		Fever		Local Redness		Local Induration		Other Symptoms	
		AEFI Cases	%	AEFI Cases	%	AEFI Cases	%	AEFI Cases	%
Age Group	6–35 months	585	28.13	83	3.99	42	2.02	241	11.59
	3–17 years	832	40.00	299	14.38	142	6.83	397	19.09
	18–49 years	46	2.21	112	5.38	65	3.13	53	2.55
	≥50 years	35	1.68	36	1.73	17	0.82	29	1.39
χ^2^		264.966		233.759		136.589		0.609	
*p*		0.000		0.000		0.000		0.894	
Sex	Male	741	35.63	235	11.30	112	5.38	343	16.49
	Female	757	36.39	295	14.18	154	7.40	377	18.13
χ^2^		7.504		3.024		3.687		0.001	
*p*		0.006		0.082		0.055		0.977	
Vaccine Type	Trivalent (split, 6–35 months)	549	26.39	82	3.94	42	2.02	226	10.87
	Trivalent (split, ≥3 years)	199	9.57	83	3.99	40	1.92	82	3.94
	Freeze-dried live (nasal spray)	38	1.83	1	0.05	1	0.05	14	0.67
	Quadrivalent (split, 6–35 months)	5	0.24	1	0.05	0	0.00	2	0.10
	Quadrivalent (split, ≥3 years)	644	30.96	337	16.20	167	8.03	366	17.60
	Quadrivalent (split, ≥6 months)	63	3.03	26	1.25	16	0.77	30	1.44
χ^2^/Fisher		96.501		104.026		46.581 *		7.542	
*p*		0.000		0.000		0.000		0.183	
Time Interval	≤1 day	1379	66.30	458	22.02	237	11.39	653	31.39
	2–3 days	100	4.81	64	3.08	23	1.11	57	2.74
	4–7 days	15	0.72	8	0.38	5	0.24	5	0.24
	8–14 days	4	0.19	0	0.00	0	0.00	4	0.19
	≥15 days	0	0.00	0	0.00	1	0.05	1	0.05
Fisher		23.967 *		14.188 *		6.514 *		7.896 *	
*p*		0.000		0.003		0.147		0.064	

Note: AEFIs: adverse events following immunization; * Fisher’s exact test.

## Data Availability

The data generated and analyzed during the current study are available from the corresponding author upon reasonable request.

## Supplementary Materials

The following supporting information can be downloaded at: https://www.mdpi.com/article/10.3390/vaccines13111154/s1, Figure S1: Seasonal Distribution of AEFI IR after Influenza Vaccination in Jiangsu Province, 2019–2023.
