# Supplementary material for: Active Surveillance of Adverse Events Following Influenza Immunization in Jiangsu Province, China: A 2019–2023 Retrospective Study
Source: Vaccines (Basel). 2025 Nov 11;13(11):1154. doi: 10.3390/vaccines13111154 (PMC12656768; doi:10.3390/vaccines13111154)
Supplement: Supplementary file 1 [file vaccines-13-01154-s001.zip › vaccines-3916304-supplementary.pdf]

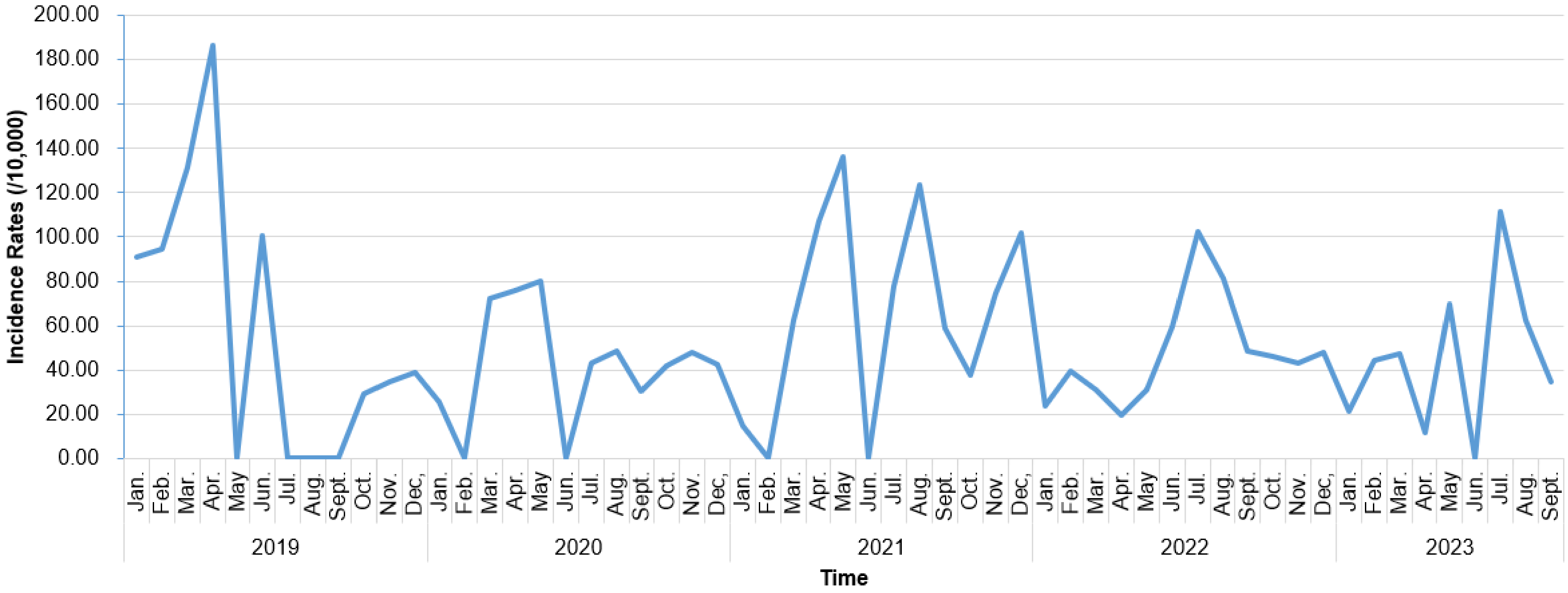

Supplementary Figure S1. Seasonal Distribution of AEFI IR after Influenza Vaccination in Jiangsu Province, 2019-2023
